# Validation of Analytical Methodology for Glyphosate Determination and Degradation Assessment with the Silver Arsenate Photocatalyst

**DOI:** 10.3390/ijerph23030284

**Published:** 2026-02-25

**Authors:** Amanda Oliveira Mourão, Mayra Soares Santos, Thuanny Souza Xavier Santos, Márcia Cristina da Silva Faria, Elton Santos Franco, Caio César de Souza Alves, Sandra Bertelli Ribeiro de Castro, Márcio César Pereira, Luiz Carlos Alves de Oliveira, Jairo Lisboa Rodrigues

**Affiliations:** 1 Instituto de Ciência, Engenharia e Tecnologia, Universidade Federal dos Vales do Jequitinhonha e Mucuri, Teófilo Otoni 39803-371, MG, Brazil; mayra.soares@ufvjm.edu.br (M.S.S.); thuanny.souza.xavier@gmail.com (T.S.X.S.); marcia.faria@ufvjm.edu.br (M.C.d.S.F.); elton.santos@ufvjm.edu.br (E.S.F.); caio.alves@ufvjm.edu.br (C.C.d.S.A.); bertelli.ribeiro@ufvjm.edu.br (S.B.R.d.C.); marcio.pereira@ufvjm.edu.br (M.C.P.); jairo.rodrigues@ufvjm.edu.br (J.L.R.); 2 Departamento de Química, Universidade Federal de Minas Gerais, Belo Horizonte 31270-901, MG, Brazil; lcao.ufmg@gmail.com

**Keywords:** herbicide, derivatization, liquid chromatography, photodegradation, bioassays

## Abstract

**Highlights:**

**Public health relevance—How does this work relate to a public health issue?**

- The extensive agricultural application of glyphosate leads to water resource contamination, presenting cytotoxic and genotoxic risks to human health and non-target organisms.
- Effective monitoring and remediation strategies are crucial to mitigate the environmental and public health impacts of herbicide residues in aquatic ecosystems.

**Public health significance—Why is this work of significance to public health?**

- This study validates a High-Performance Liquid Chromatography (HPLC-FLD) method that is sensitive, precise, and accurate for quantifying glyphosate, ensuring reliable water quality monitoring.
- The Silver Arsenate (Ag_3_AsO_4_) photocatalyst demonstrates 99.46% removal of glyphosate under visible light, offering an efficient solution for pollutant elimination.

**Public health implications—What are the key implications or messages for practitioners, policy makers and/or researchers in public health?**

- The validated methodology offers a robust, cost-effective tool for assessing water compliance with regulatory herbicide limits.
- Ag_3_AsO_4_ exhibits high stability and reusability with no observed cytotoxicity, highlighting its potential as a sustainable material for advanced water treatment technologies.

**Abstract:**

Given the extensive use and toxicity of glyphosate, this study aimed to optimize and validate a high-efficiency liquid chromatography method with fluorescence detection for its quantification in water, evaluate its photocatalytic degradation using Ag_3_AsO_4_, and assess biological toxicity via the *Allium cepa* test, the MTT assay, and nitric oxide quantification in RAW264.7 cells. The analytical method was successfully validated, exhibiting a correlation coefficient of 0.99976 and limits of detection and quantification of 0.0314 μg L^−1^ and 0.1048 μg L^−1^, respectively, with coefficients of variation below 9.05% and recovery rates between 93.84 and 99.41%. Regarding degradation, the Ag_3_AsO_4_ photocatalyst achieved a glyphosate removal rate of 99.46% within 60 min under visible light. Furthermore, the material demonstrated high stability and reusability, with only a 5.03% decrease in degradation efficiency after three consecutive cycles. Biological assays indicated that glyphosate possesses cytotoxic and genotoxic potential in the analyzed cells. These findings confirm the effectiveness of Ag_3_AsO_4_, highlighting its potential as a candidate material for environmental remediation, although further studies on metal leaching are required.

## 1. Introduction

Agriculture has expanded and modernized its activities over the years to meet the growing demand for food driven by continuous population growth. These factors drive the use of pesticides in crops for pests control and, consequently, the genetic improvement of crops to ensure resistance to these compounds. Pesticides increase crop productivity and quality, maximize economic return [1,2].

Herbicides are a class of pesticides widely used in agriculture for weed control. Glyphosate is the most widely marketed herbicide worldwide, characterized as a broad-spectrum, post-emergence organophosphoric compound that is non-selective for unwanted plants. Its mechanism of action involves the inhibition of enzymes that interfere with the synthesis of amino acids, consequently inhibiting the growth of plants that absorb glyphosate [3,4]. Its widespread use results in soil and water contamination, causing adverse effects on human health and non-target organisms exposed to the environment [4,5,6].

Chromatographic methods for glyphosate analysis reported in the literature include ion chromatography (IC) [7], gas chromatography–mass spectrometry (GC-MS) [8], gas chromatography–tandem mass spectrometry (GC-MS/MS) [9], flame photometric detection (GC-FPD) [10], high-performance liquid chromatography–mass spectrometry (HPLC-MS) [11,12], high-performance liquid chromatography–tandem mass spectrometry (HPLC-MS/MS) [13], high-performance liquid chromatography–ultraviolet (HPLC-UV) [14,15], and high-performance liquid chromatography–fluorescence detection (HPLC-FLD) [16,17].

Liquid chromatography is the most commonly used technique for the analysis of this herbicide due to its ionic character and low volatility. However, for ultraviolet or fluorescence detection, a derivatization process is necessary due to the lack of chromophores or fluorophores in its structure. The most commonly used derivatizing agents are 9-fluorenylmethyl chloroformate (FMOC-Cl), 4-chloro-7-nitrobenzofurazan (NBD-Cl), 2,2-dihydroxyindane-1,3-dione (ninhydrin), l-fluoro-2,4-dinitrobenzene (DNP), o-phthalaldehyde-mercaptoethanol (OPA), and p-toluenesulfonyl chloride (TsCl). Pre-column derivatization with FMOC-Cl is commonly used as it reacts with the secondary amine of glyphosate, producing stable and highly fluorescent derivatives [17,18].

The association of glyphosate with harmful effects on living organisms has sparked discussions regarding its toxic potential by national and international regulatory agencies. Biological tests are widely used to determine toxicity levels of contaminants in aquatic matrices. There are different types of bioassays, which vary according to the purpose and organism used, such as the *Allium cepa* test, the MTT assay, and the quantification of nitric oxide in murine macrophage cell lines. Among the advantages of applying these tests are their high efficiency, fast execution, and low cost [19,20,21].

An alternative for glyphosate degradation is the use of advanced oxidation processes (AOPs), since this contaminant is not removed by conventional water treatment processes. AOPs have shown satisfactory results in the degradation of organic compounds in water and are based on the formation of hydroxyl radicals (•OH), which are highly oxidizing species capable of degrading and mineralizing organic pollutants into non-toxic compounds, such as CO_2_ and H_2_O [22,23,24,25].

Among AOPs, heterogeneous photocatalysis stands out as a process that involves redox reactions induced by radiation on the surface of catalysts. Ag-based photocatalysts have shown promise in the degradation of organic compounds due to their narrow band gaps, which allow for effective excitation under visible light irradiation [26,27,28]. Among these, silver arsenate (Ag_3_AsO_4_) was selected for this study due to its distinct advantages, including a facile and reproducible synthesis method and high photooxidative capability. Furthermore, previous studies by our research group have demonstrated the robustness of this material in degrading various organic contaminants, motivating its specific evaluation for glyphosate remediation [29].

Hott et al. [29] and Tang et al. [30] reported the use of Ag_3_AsO_4_ in the degradation of rhodamine B, with a full removal rate of the dye due to its high oxidation capacity. Additionally, after the reaction, the photocatalyst can be easily recovered and reused in subsequent degradation processes. To date, however, based on a comprehensive search in Scopus, Web of Science, and Google Scholar databases (accessed in January 2026), there are no reports in the literature describing the application of Ag_3_AsO_4_ for the photocatalytic degradation of the herbicide glyphosate.

Although arsenic compounds are generally associated with toxicity, it is important to distinguish soluble species from stable solid-state photocatalysts. Ag_3_AsO_4_ is characterized by its high structural stability and an extremely low solubility product constant (Ksp ≈ 10^−22^), which significantly mitigates the risk of releasing free arsenate ions into the aqueous medium under neutral pH conditions. This chemical stability makes it a promising candidate for environmental applications, provided that post-treatment separation is effectively managed.

Based on these aspects, this work aims to develop and validate a fast, low-cost, and effective method for the determination of glyphosate in water samples, performing pre-column derivatization with FMOC-Cl. Subsequently, the degradation of this compound with the synthesized Ag_3_AsO_4_ photocatalyst was evaluated by HPLC-FLD analysis, as well as the evaluation of the toxicity of glyphosate and Ag_3_AsO_4_ through the *Allium cepa* test, MTT assay, and quantification of nitric oxide in RAW264.7 cells, as a proposal for environmental monitoring and water quality improvement regarding this contaminant.

## 2. Materials and Methods

### 2.1. Reagents, Solutions, and Equipment

All reagents used were of analytical grade. Pestanal^®^ glyphosate standard (purity ≥ 98%) was obtained from Sigma-Aldrich (St. Louis, MO, USA). A standard stock solution of glyphosate at a concentration of 50 mg L^−1^ was prepared in ultrapure water (resistivity 18.2 MΩ cm) obtained by the Barnstead Nanopure system (Thermo Scientific, Waltham, MA, USA). The working solutions were prepared by diluting the glyphosate standard stock solution. A stock solution of the derivative agent 9-fluorenylmethyl chloroformate (purity ≥ 98%) at a concentration of 10 g L^−1^ was prepared in acetonitrile (for HPLC, grade gradient, purity ≥ 99.9%), both obtained from Sigma-Aldrich (St. Louis, MO, USA). The glyphosate and FMOC-Cl solutions were stored at 4 °C.

Borate buffer was prepared with sodium tetraborate decahydrate (BioXtra, purity ≥ 99.5%) obtained from Sigma-Aldrich (St. Louis, MO, USA) and adjusted to pH 9 with 0.1 mol L^−1^ sodium hydroxide (Synth, São Paulo, Brazil). Phosphate buffer (0.05 mol L^−1^, pH 5.5) used in the mobile phase was prepared with potassium phosphate monobasic (purity ≥ 99.5%) obtained from Sigma-Aldrich (St. Louis, MO, USA), and the pH was adjusted with potassium hydroxide 6 mol L^−1^ (Dinâmica Química, São Paulo, Brazil). Silver nitrate (Plat-Lab, São Paulo, Brazil) and sodium arsenate dibasic heptahydrate (Sigma-Aldrich, St. Louis, MO, USA) were used in silver arsenate synthesis (Ag_3_AsO_4_).

### 2.2. Derivatization of Glyphosate

The derivatization procedure was based on the method described by Le Bot et al. [31], who employed a mixture of 3 mL of sample, 0.5 mL of 0.05 mol L^−1^ borate buffer, and 500 µL of 1 g L^−1^ FMOC-Cl solution. The reaction was performed under agitation at room temperature for 1 h. In the present study, modifications were introduced to optimize reaction conditions. Optimal parameters were selected based on direct evaluation of integrated peak areas, identifying conditions that yielded the maximum chromatographic response. A provisional reaction time of 30 min was initially employed to optimize borate buffer and FMOC-Cl concentrations. 2.5 mL of the glyphosate standard was transferred to a 15 mL polypropylene tube, followed by the addition of 500 μL of borate buffer (0.04, 0.05, and 0.06 mol L^−1^) at pH 9 and 500 μL of FMOC-Cl (0.25, 0.5, 1, and 1.5 g L^−1^). After reagent addition, the mixture was vortexed for 1 min to ensure immediate homogenization. Subsequently, the reaction was allowed to proceed under static conditions at room temperature. To determine the optimal derivatization time, different intervals were evaluated (5, 15, 30, 45, 60, and 80 min). Subsequently, the mixture was filtered into a vial using a 0.22 μm regenerated cellulose membrane syringe filter and immediately analyzed by HPLC-FLD.

### 2.3. Analytical Validation

The analytical method proposed in this paper was validated according to Anvisa [32] and INMETRO [33]. Validated parameters were selectivity, linearity, precision, accuracy, limit of detection, and limit of quantification.

Selectivity was assessed by comparing chromatograms obtained from the analysis of the glyphosate-free standard matrix with the matrix containing the analyte of interest at 50 μg L^−1^. Linearity was verified by a calibration curve prepared with a standard glyphosate solution at concentrations of 1, 5, 12.5, 25, 50, and 100 μg L^−1^ in triplicate. The precision study was carried out based on repeatability and intermediate precision (performed on two consecutive days), expressed as relative standard deviation (RSD), for concentrations of 1, 5, and 12.5 μg L^−1^, with each solution prepared in triplicate. Accuracy was assessed through recovery trials by spiking samples with known amounts of analyte at concentrations of 1, 5, and 12.5 μg L^−1^, with triplicate determinations. The limit of detection (LOD) and limit of quantification (LOQ) were determined by estimation from the analytical curve, using the standard deviation value of the lowest level of the calibration curve.

### 2.4. Synthesis of Ag_3_AsO_4_

The photocatalyst silver arsenate was synthesized according to Ge et al. [26], with adaptations. A total of 0.340 g of AgNO_3_ was solubilized in 15 mL of ultrapure water in an Erlenmeyer. In another Erlenmeyer, 0.2059 g of Na_2_HAsO_4_ was dissolved in 30 mL of ultrapure water. The solution containing Na_2_HAsO_4_ was added drop by drop to the solution of AgNO_3_ under constant stirring and remained in the magnetic stirrer for 60 min at a temperature of 25 °C. The brown precipitate formed was transferred to 50 mL Falcon tubes, centrifuged at 3600 rpm for 3 min, washed three times with ultrapure water, and the fourth time with 99% ethyl alcohol, and then dried at 55 °C for 6 h.

### 2.5. Characterization of Ag_3_AsO_4_

The synthesized Ag_3_AsO_4_ was characterized by scanning electron microscopy (SEM), energy-dispersive spectroscopy (EDS), X-ray diffraction (XRD), and diffuse reflectance spectroscopy (DRS). The surface morphology of the synthesized compound was examined using a HITACHI TM-3000 Scanning Electron Microscope (Tokyo, Japan) with a Swift ED 3000 X-ray microanalysis detector from Oxford Instruments (Tubney Woods, Abingdon, UK). SEM images and EDS information were obtained using an acceleration voltage of 15 kV. The XRD pattern was taken in the range of 20–90° 2θ with a Rigaku Geigerflex (Rigaku, Tokyo, Japan) diffractometer equipped with a graphite diffracted beam monochromator, using CuKα radiation. The optical properties were determined using a Shimadzu UV-3600 spectrometer (Shimadzu, Kyoto, Japan). The DRS data were recorded in the range from 200 to 900 nm.

### 2.6. Photodegradation of Glyphosate

The study of glyphosate photodegradation parameters in water samples was carried out to determine the irradiation time, pH, and amount of Ag_3_AsO_4_ catalyst that resulted in the higher removal rate. In a 50 mL polypropylene beaker, 10 mL of glyphosate standard solution (50 μg L^−1^) and varying amounts of Ag_3_AsO_4_ photocatalyst (5, 10, and 20 mg) were added. The pH (5, 7, and 9) was adjusted with HCl 0.1 mol L^−1^ and NaOH 0.1 mol L^−1^. The material was kept in suspension by magnetic stirring for 30 min in the dark to establish adsorption/dessorption equilibrium between the analyte and catalyst. A 2 mL aliquot was removed, filtered into a vial with a 0.22 μm regenerated cellulose membrane syringe filter, and analyzed by HPLC-FLD. The catalyst suspension was then irradiated with white LED light (λ > 450 nm, 0.55 mW cm^−2^) positioned at a distance of 3 cm from the solution surface. A control experiment (photolysis) was also performed under identical conditions in the absence of the photocatalyst. During the reaction, a 2 mL aliquot was removed at intervals of 0 to 60 min, filtered into a vial with 0.22 μm regenerated cellulose membrane syringe filter, and analyzed by HPLC-FLD.

Additionally, Ag_3_AsO_4_ reuse experiments were performed to evaluate photocatalyst stability after the first, second, and third cycles, using 10 mg of material, an irradiation time of 20 min, and pH 7. After each cycle, the photocatalyst was recovered by centrifugation at 3600 rpm for 3 min, washed three times with ultrapure water, and once with 99% ethyl alcohol, and dried at 55 °C for 6 h before starting a new photocatalytic degradation cycle.

### 2.7. Bioassays

#### 2.7.1. *Allium cepa* Test

The cytotoxic, genotoxic, and mutagenic potential of glyphosate and the synthetic silver arsenate ware evaluated using the *Allium cepa* test according to Grant [34] and Leme and Marin-Morales [35], with modifications.

*Allium cepa* seeds (100 per dish) were germinated at room temperature (21 ± 4 °C) in Petri dishes covered with filter paper soaked in water sample prepared with Ag_3_AsO_4_ at 0.1% (*w*/*v*) and in ultrapure water samples prepared with glyphosate standard at concentrations of 65 and 280 μg L^−1^, before and after degradation with Ag_3_AsO_4_. The negative control was performed with Milli-Q water and the positive control with methyl methanesulfonate (MMS) at 0.4 mmol L^−1^. After roots reached a length of about 1.5 cm (5 to 7 days), they were cut and fixed in Carnoy 3:1 solution (ethanol: acetic acid, *v*/*v*) in Eppendorf tubes for 6 h. Then, roots were fixed in 70% alcohol and kept for 24 h. They were then transferred to a new 70% alcohol solution and stored at 4 °C until analysis.

Slides with meristematic root cells were prepared according to Mondin and Aguiar-Perecin [36], with optimizations to enhance chromosomal definition. The main modifications included adjusting the hydrolysis time, staining duration, and counter-stain concentration. Roots preserved in 70% alcohol were washed three times with distilled water for 5 min each. Then, they were subjected to acid hydrolysis with 1 mol L^−1^ HCl solution at 60 °C for 11 min, washed three times with distilled water for 5 min, and excess water was removed with filter paper. After acid hydrolysis, roots were stained with Schiff’s reagent for 2 h in the dark and washed with distilled water until the excess reagent was removed. Slides were prepared with the meristematic regions cut and placed on slides with a drop of 2% acetic carmine. After 5 min, each slide was covered with a coverslip and slightly smashed for analysis under an optical microscope (FLUO-3, Bel Engineering, Monza, Italy). To evaluate cell damage, 10 slides were prepared for each sample and 500 cells per slide were analyzed, totaling 5000 cells per treatment.

The cytotoxic potential was determined using the mitotic index (IM), i.e., the number of dividing cells, calculated as the ratio between the number of mitotic cells and the total number of cells, expressed as a percentage. Genotoxic effects were assessed by observation and quantification of chromosomal aberrations in meristematic cells. Mutagenic potential was determined based on the number of micronucleated cells in meristematic regions.

Statistical analyses were performed using GraphPad Prim software version 10.2.2. Analysed groups were compared using the Mann–Whitney test. Differences between controls and treatments were considered statistically significant when *p* < 0.05. Experimental data were expressed as mean ± standard deviation of independent experiments.

#### 2.7.2. Cell Viability Evaluation by the MTT Assay

For the MTT assay and quantitative determination of nitric oxide, the RAW264.7 murine macrophage cell line was used and kept in culture flasks containing RPMI-1640 medium supplemented with 0.002 mol L^−1^ L-glutamine, 100 μg mL^−1^ streptomycin and penicillin, and 5% fetal bovine serum in a humidified atmosphere of 5% CO_2_ at 37 °C. After reaching 90% confluence, the flasks were scraped, and the cells were plated in 96-well plates at 2 × 10^5^ mL^−1^ cells. Macrophages were cultured for 48 h in the presence of Ag_3_AsO_4_ at a concentration of 0.1% (*w*/*v*) or a standard glyphosate solution at concentrations of 65 and 280 μg L^−1^, before and after degradation with Ag_3_AsO_4_.

Cell viability was determined using the MTT [3-(4,5-dimethylthiazol-2-yl)-2,5-diphenyltetrazolium bromide) assay. After 48 h of culture, the supernatants were removed, and the cells were incubated with 100 μL of supplemented RPMI and 10 μL of MTT at a concentration of 5 mg mL^−1^ for 4 h in a humidified atmosphere with 5% CO_2_ at 37 °C. After this period, the supernatant was removed and the formazan crystals formed were dissolved by adding 100 μL of dimethyl sulfoxide (DMSO) to each plate under slight agitation. The optical density was measured at 570 nm (EZ Read 2000, Biochrom, Cambridge, UK).

The results presented are representative of three independent experiments and are shown as mean ± standard error. Statistical significance was analyzed using Student’s *t*-test. Differences were considered significant when *p* < 0.05.

#### 2.7.3. Nitric Oxide Measurement

The nitric oxide concentration was determined indirectly by the nitrite concentration present in the supernatant after 48 h using the Griess method. A 100 μL aliquot of supernatant from each well of the stimulated culture was transferred to 96-well plates, and an equal volume of Griess reagent (1% sulfanilamide, 0.1% N-(1-Naphthyl)ethylenediamine, and 5% H_3_PO_4_) was added. Plates were stimulated with 10 μg mL^−1^ lipopolysaccharide (LPS) and 9 ng mL^−1^ IFN-γ. Untreated and unstimulated cells were used as the negative control, and stimulated and untreated cells were used as the positive control. The nitric oxide concentration was determined by comparing the nitrite production with a calibration curve constructed using different concentrations of a sodium nitrite standard solution. The optical density was measured at 540 nm (EZ Read 200, Biochrom).

### 2.8. Instruments and Chromatographic Conditions

Chromatographic analyses were performed using a Perkin Elmer Flexar HPLC system (Waltham, MA, USA) equipped with a quaternary pump, an autosampler, and a fluorescence detector. System control, data acquisition, and processing were performed using Chromera software version 4.1. Separation was performed using a PerkinElmer HyperSelect ODS C18 reversed phase column (150 mm × 4.6 mm, 5 μm i.d.). The mobile phase consisted of 0.05 mol L^−1^ phosphate buffer (pH 5.5): acetonitrile (60:40), in isocratic elution mode. The column oven temperature was set to 25 °C and the injection volume was 20 μL. The total run time was 15 min at a flow rate of 0.8 mL min^−1^. The excitation and emission wavelengths used for glyphosate detection were 265 nm and 315 nm, respectively.

## 3. Results and Discussion

### 3.1. Optimization of Derivatization Parameters

#### 3.1.1. Effect of FMOC-Cl Concentration

The molar ratio between the analyte and the derivatization reagent influences the formation of the derivatized product. For the reaction to proceed effectively, an excess of FMOC-Cl must be added to the sample, as the derivatization reagent reacts not only with analytes but also with other amines, amino acids, and water present in the sample, forming by-products such as FMOC-OH, which do not interfere with glyphosate determination [17,37].

**Figure 1:**
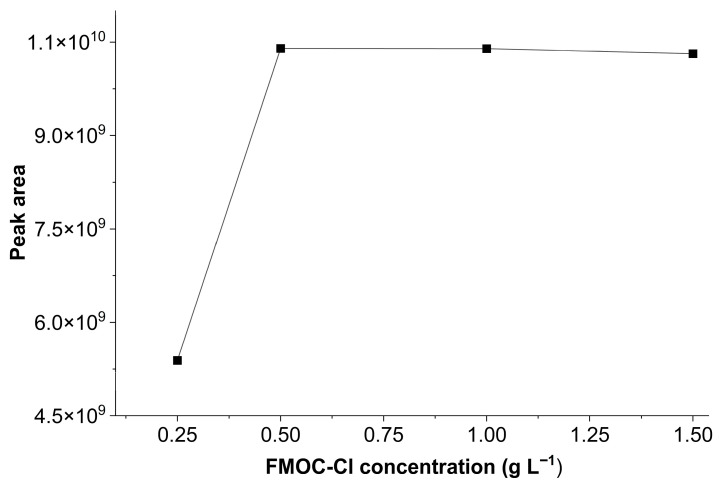
Effects of FMOC-Cl concentration (0.25, 0.5, 1, and 1.5 g L^−1^) on the chromatographic peak area of the glyphosate standard (50 μg L^−1^), using 0.05 mol L^−1^ borate buffer and a reaction time of 30 min.

Thus, the influence of FMOC-Cl concentration on the derivatization reaction was investigated. Derivatization reagent concentrations varied at 0.25, 0.5, 1, and 1.5 g L^−1^. Figure 1 shows the glyphosate response, evaluated by chromatographic peak area, for different FMOC-Cl concentrations. A lower glyphosate response was observed at 0.25 g L^−1^, while no significant changes in analyte peak area were observed at other concentrations. Therefore, to obtain an optimal analytical response and reduce derivatization agent excess, a concentration of 0.5 g L^−1^ was selected for subsequent experiments.

#### 3.1.2. Effect of Borate Buffer Concentration

Proper borate buffer concentration in the derivatization reaction is necessary to maintain buffering capacity and stabilize the basic medium so that the reaction proceeds effectively [17].

**Figure 2:**
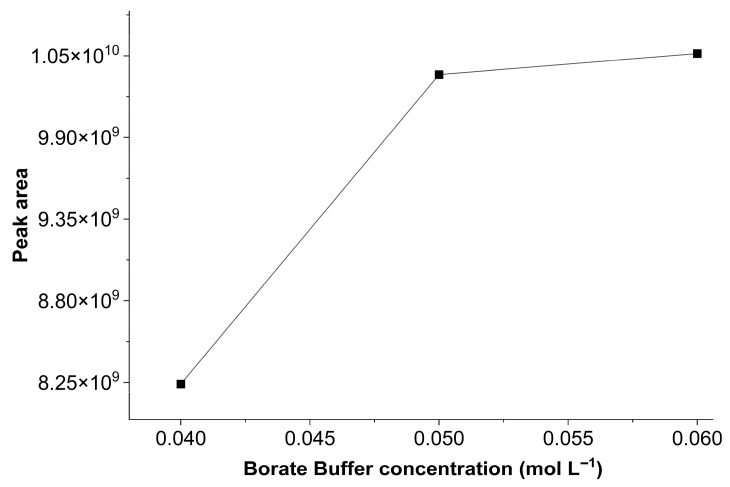
Effects of borate buffer concentration (0.04, 0.05, and 0.06 mol L^−1^) on the chromatographic peak area of the glyphosate standard (50 μg L^−1^), using 0.5 g L^−1^ FMOC-Cl and a reaction time of 30 min.

Thus, the influence of borate buffer concentration on the derivatization reaction was tested. Concentrations tested were 0.04, 0.05, and 0.06 mol L^−1^, and results are shown in Figure 2. It was found that at 0.04 mol L^−1^, the derivatization reaction did not occur effectively. However, the response to glyphosate increased at buffer concentrations of 0.05 and 0.06 mol L^−1^, with no significant differences between the two areas. Increasing the buffer concentration promotes the reactivity of the amine group, which loses hydrogen atom and binds to the carboxyl group of FMOC-Cl in the basic medium, favoring the derivatization process [38]. Therefore, a borate buffer concentration of 0.05 mol L^−1^ was chosen for the subsequent tests.

#### 3.1.3. Effect of Derivatization Time

**Figure 3:**
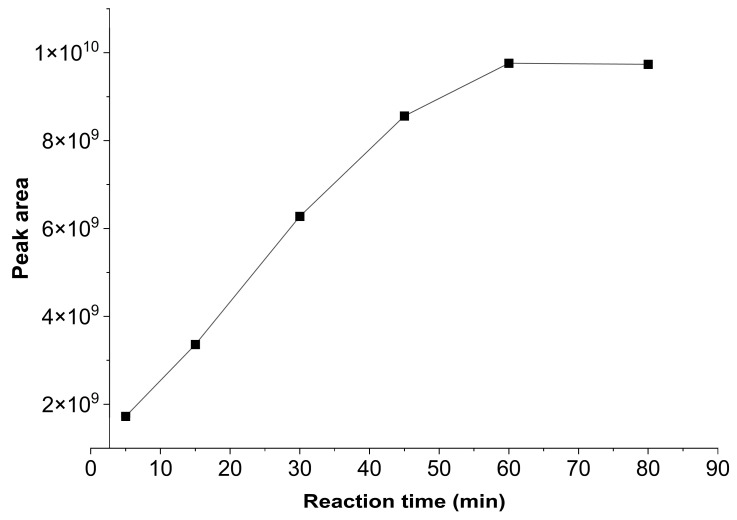
Effects of derivatization time (5, 15, 30, 45, 60, and 80 min) on the chromatographic peak area of the glyphosate standard (50 μg L^−1^), using 0.5 g L^−1^ FMOC-Cl and 0.05 mol L^−1^ borate buffer.

The time in which the derivatization reaction takes place is an important factor in ensuring that analytes are completely converted into derivatized and detectable forms. Therefore, the equilibrium time required for the derivatization reaction was evaluated at 5, 15, 30, 45, 60, and 80 min. Figure 3 shows the evaluated derivatization times and respective chromatographic peaks areas for glyphosate.

Results show that the glyphosate peak area gradually increased with increasing reaction time. However, times of 60 and 80 min showed no significant differences in glyphosate response. Therefore, to achieve better derivatization reaction yield and higher analytical productivity, a reaction time of 60 min was established for the further tests.

### 3.2. Analytical Validation

#### 3.2.1. Selectivity

**Figure 4:**
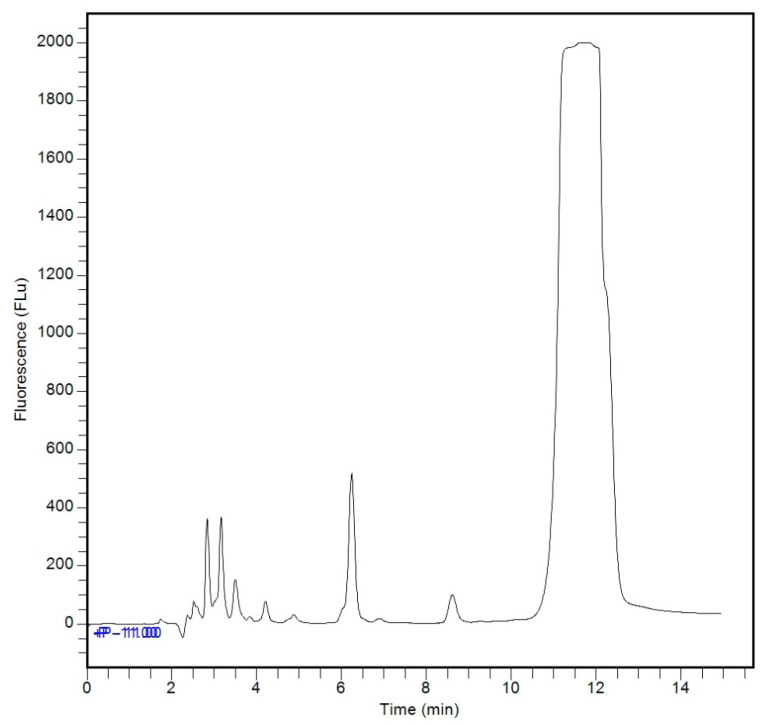
HPLC-FLD chromatogram of the analyte-free matrix with FMOC-Cl and borate buffer, with HPLC-FLD analysis.

**Figure 5:**
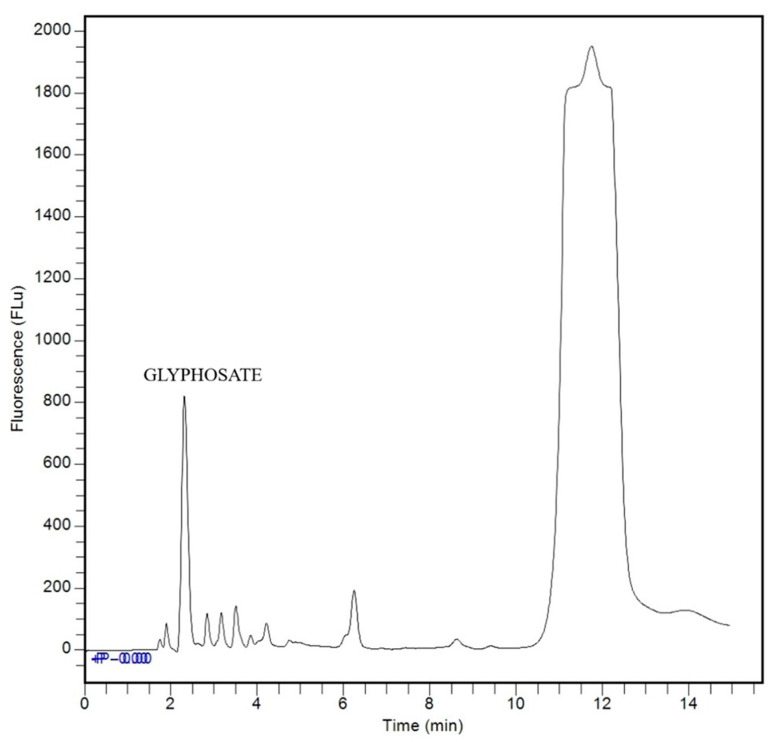
HPLC-FLD chromatogram of the derivatized glyphosate standard at a concentration of 50 μg L^−1^.

Method selectivity was evaluated by comparing chromatograms of an analyte-free standard matrix (blank) (Figure 4), which contained only FMOC-Cl and borate buffer, with a matrix containing the glyphosate standard (Figure 5), analyzed after the derivatization reaction. Chromatograms analysis demonstrated method selectivity, as no interfering peaks were observed at the glyphosate retention time (2.3 min), indicating that sample matrix components do not interfere with the measurement.

#### 3.2.2. Linearity

**Figure 6:**
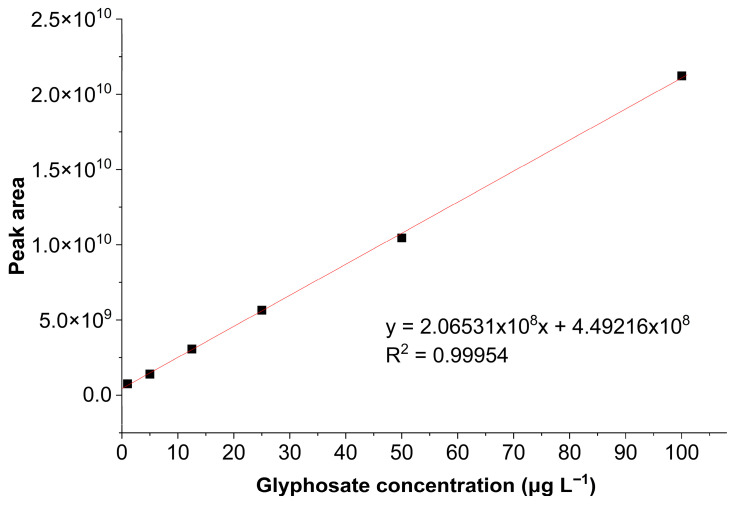
Calibration curve for the glyphosate standard.

The calibration curve for glyphosate in the range of 1–100 μg L^−1^ (Figure 6) was established, and curve parameters are listed in Table 1.

The analytical curve exhibited a linear relationship, confirmed by the correlation coefficient, which showed a satisfactory value above the minimum required by Anvisa [32]. Thus, the analytical method showed linearity within the working range studied, since the correlation coefficient was greater than 0.99.

#### 3.2.3. Precision

Repeatability (intraday) and intermediate precision (interday), expressed as the relative standard deviation (RSD), were evaluated at concentrations of 1, 5, and 12.5 μg L^−1^, and results are shown in Table 2.

The relative standard deviations calculated for the precision tests were less than 6.28% and 9.04% for repeatability and intermediate precision, respectively. Since these values are below the 15% limit recommended by INMETRO [33], the proposed method is considered precise for glyphosate determination.

#### 3.2.4. Accuracy

Accuracy was assessed by recovery tests at concentrations of 1, 5, and 12.5 μg L^−1^. Measured concentrations and analyte recovery results are shown in Table 3.

Method accuracy was demonstrated by satisfactory recoveries ranging from 93.84% to 99.41%, falling within the 80 to 110% range recommended by INMETRO [33].

#### 3.2.5. Limit of Detection and Limit of Quantification

The analytical curve was used to estimate the limit of detection and limit of quantification, as this method provides better results at the trace level. The standard deviation of the lowest level of the calibration curve (1 μg L^−1^), was used to calculate both parameters, as the blank value does not generate a signal.

Results for limit of detection and limit of quantification were 0.0314 μg L^−1^ and 0.1048 μg L^−1^, respectively, indicating that the analytical method has a high degree of reliability in detecting and quantifying low glyphosate concentrations.

### 3.3. Characterization of Ag_3_AsO_4_

**Figure 7:**
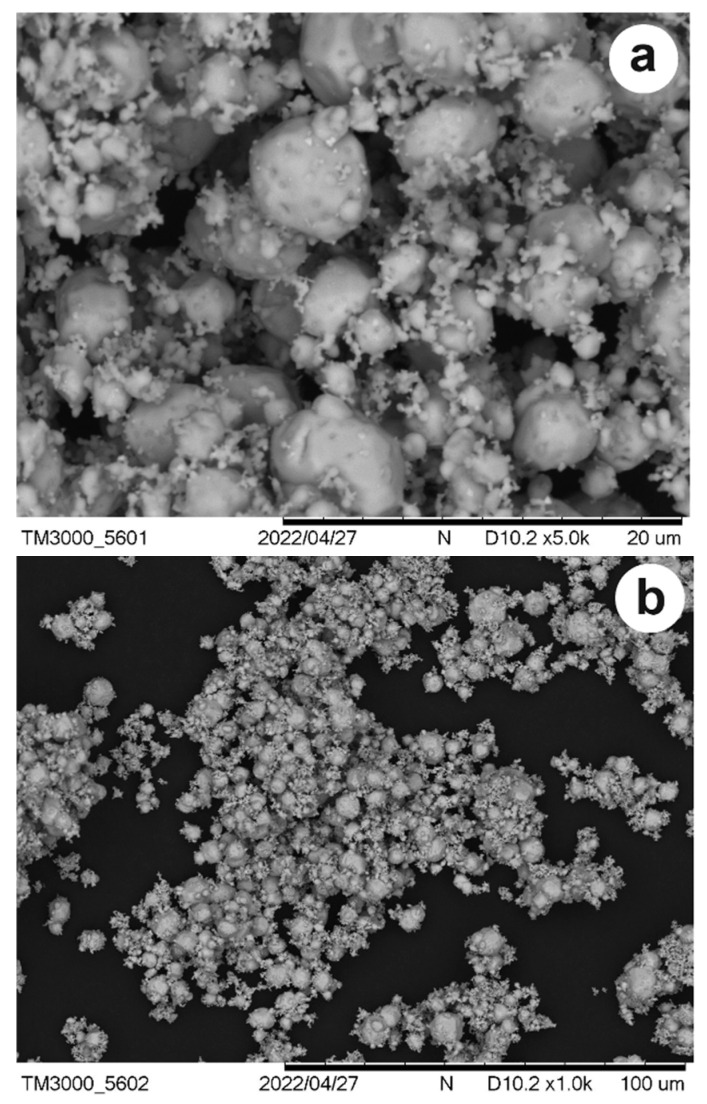
Images of Ag_3_AsO_4_ particles taken by SEM at magnifications of (**a**) 5kx and (**b**) 1kx.

Figure 7 shows SEM images of Ag_3_AsO_4_ particles. It can be observed that the silver arsenate exhibits irregular shapes with average particle sizes between 25 and 100 μm. EDS analysis allowed for the determination of the percentage elemental composition of Ag_3_AsO_4_. It was found that the synthesized material is composed of 12.57% As, 19.66% O, and 67.77% Ag by weight.

**Figure 8:**
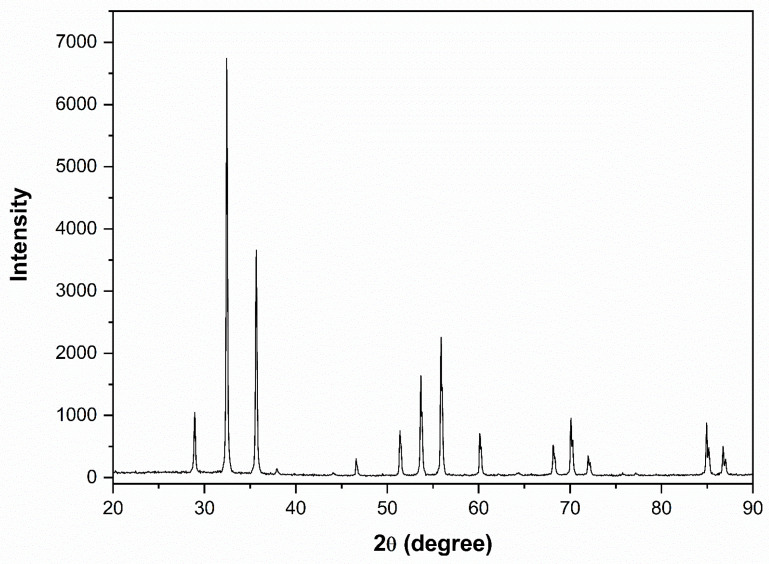
Powder XRD pattern of synthetic Ag_3_AsO_4_. The three most intense diffraction peaks correspond to the (210), (211), and (321) crystallographic planes, confirming the structure of the synthesized material.

XRD analysis was performed to identify the composition and crystalline structure of the compound. Figure 8 shows the data obtained by XRD for the synthesized Ag_3_AsO_4_ sample. All diffraction peaks can be indexed to the cubic phase of Ag_3_AsO_4_ (JCPDS card number 1–103), and no impurity peaks were detected.

**Figure 9:**
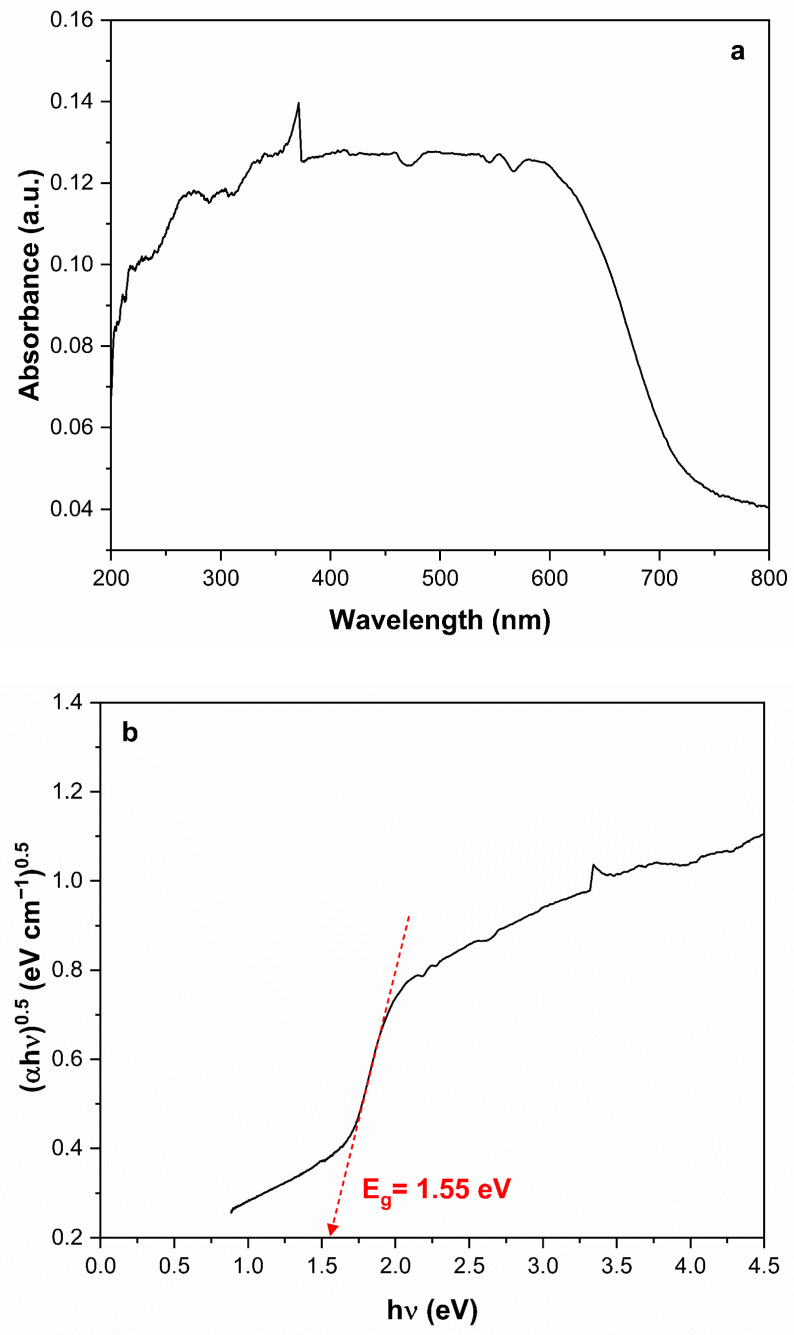
(**a**) UV-Vis DRS spectrum and (**b**) the corresponding Tauc plot for the band gap determination of Ag_3_AsO_4_.

Figure 9a shows the UV-Vis DRS spectrum of Ag_3_AsO_4_. It can be observed that Ag_3_AsO_4_ absorbs visible light in the range of 200 to 700 nm. The band gap energy of Ag_3_AsO_4_ was determined using the Tauc model: $\alpha h\nu ={A(h\nu -E_{g})}^{n}$, where α, is the linear absorption coefficient, h is Planck’s constant, ν is the light frequency, A is the proportionality constant, E_g_ is the optical bandgap energy, and n is transition order (*n* = 0.5 for direct transition and *n* = 2 for indirect transition). Based on Figure 9b, an optical band gap of 1.55 eV was found for Ag_3_AsO_4_. This value is very close to that reported by Tang et al. [30] and Hott et al. [29].

### 3.4. Photocatalytic Activity of Ag_3_AsO_4_

#### 3.4.1. Effect of Exposure Time to Visible Light

The photocatalytic activity and chemical stability of Ag_3_AsO_4_ were evaluated by glyphosate degradation under visible light. Prior to the photocatalytic tests, control experiments were performed to evaluate adsorption capacity (reaction in the dark) and direct photolysis (irradiation without catalyst). In both cases, a negligible decrease in glyphosate concentration was observed (less than 2%), indicating that the pollutant is stable under visible light irradiation and that no significant adsorption occurred.

**Figure 10:**
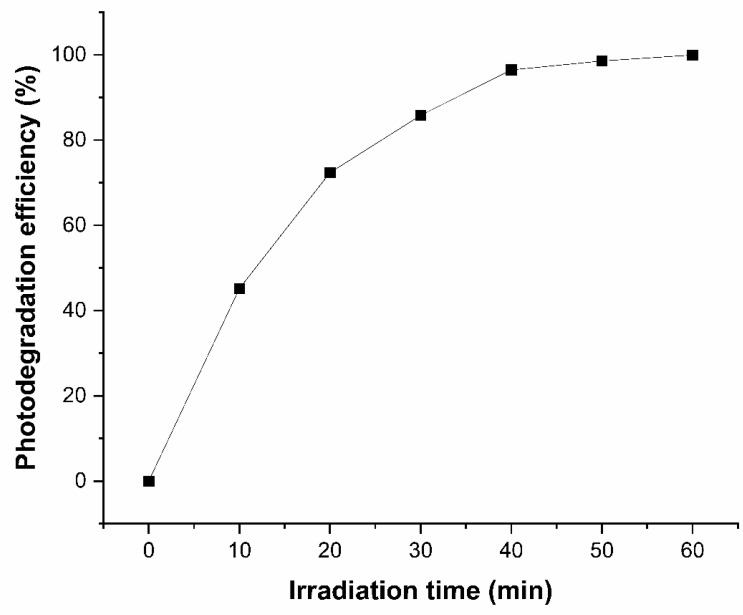
Effect of exposure time to visible light on the photodegradation efficiency of glyphosate.

Subsequently, photocatalytic degradation of glyphosate was performed, optimizing the irradiation time, amount of material, and pH. Figure 10 shows the photodegradation efficiency of glyphosate exposed to visible light over an irradiation time from 0 to 60 min. It can be observed that photodegradation efficiency increases with increasing light exposure time. After 60 min of light irradiation, the glyphosate degradation rate was 99.46%. Thus, it can be concluded that the degradation of this herbicide by the Ag_3_AsO_4_ photocatalyst was efficient after 60 min of visible light irradiation.

Table 4 compares the results of the photocatalytic activity of Ag_3_AsO_4_ in this study with other photocatalysts reported in the literature for glyphosate photodecomposition.

To the best of our knowledge, this is the first study to report the degradation of glyphosate with the Ag_3_AsO_4_ photocatalyst. Tang et al. [30] reported the excellent photocatalytic activity of Ag_3_AsO_4_ in the removal of rhodamine B and methyl orange, which is higher than that of Ag_3_PO_4_ or AgI, due to the high oxidation capacity of photogenerated holes and the high capacity to adsorb visible light. Hott et al. [29] also reported the complete degradation of rhodamine B with the Ag_3_AsO_4_ photocatalyst after 10 min of reaction time, indicating its effectiveness in removing organic compounds.

#### 3.4.2. Effect of the Amount of Ag_3_AsO_4_

**Figure 11:**
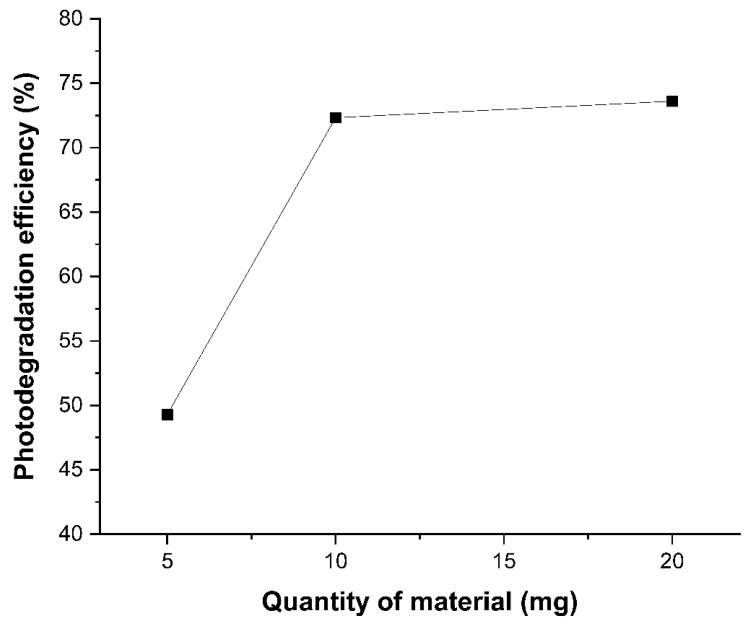
Effect of the amount of Ag_3_AsO_4_ on the efficiency of photodegradation. Irradiation time of 20 min.

The effect of the amount of Ag_3_AsO_4_ was investigated using 5, 10, and 20 mg at an irradiation time of 20 min. The relationship between photodegradation efficiency and the amount of photocatalyst is shown in Figure 11. It was found that when the amount of photocatalyst was increased from 5 to 10 mg, glyphosate photodegradation efficiency increased from 49.29% to 72.33%. Increasing the amount of catalyst to 20 mg showed no significant difference in photodegradation efficiency, which reached 73.61%. This can be explained by the shielding effect of excess material in the solution. Therefore, to achieve higher glyphosate removal efficiency with lower material consumption, the optimal amount for this study was determined to be 10 mg Ag_3_AsO_4_.

#### 3.4.3. Effect of pH

**Figure 12:**
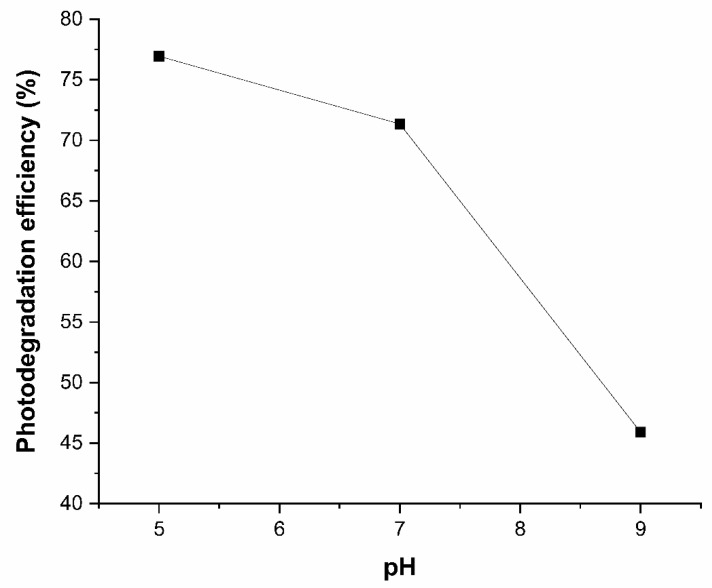
Effect of pH on glyphosate photodegradation efficiency. Irradiation time of 20 min.

The efficiency of photodegradation was investigated at pH values of 5, 7, and 9 with an irradiation time of 20 min. As shown in Figure 12, glyphosate photodegradation efficiency increased with decreasing pH, reaching 76.94% at pH 5, 71.35% at pH 7, and 41.91% at pH 9. However, there was no significant difference in efficiency between pH 5 and 7. According to Zhang et al. [44], the formation of active species such as •OH and O^2−^• is favorable under acidic conditions, which are essential for the photocatalytic degradation of organic pollutants like glyphosate. In contrast, the concentration of CO^3−^ and HCO^3−^ ions, which act as •OH scavengers, increases with increasing pH. Thus, a reduction in hydroxyl radical availability leads to decreased photodegradation efficiency.

#### 3.4.4. Stability Study of Ag_3_AsO_4_

**Figure 13:**
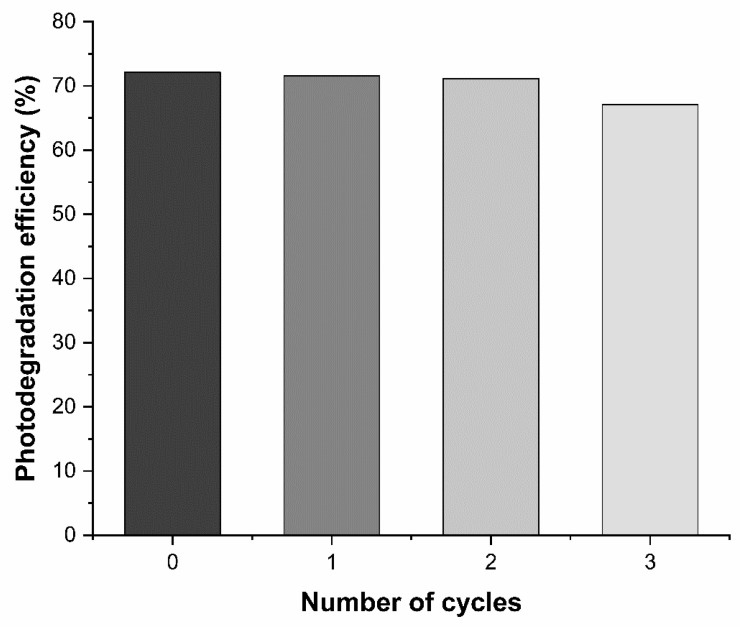
Glyphosate degradation efficiency using the Ag_3_AsO_4_ photocatalyst over three degradation cycles.

Photocatalyst stability is a crucial parameter for evaluating practical application. The stability of Ag_3_AsO_4_ was assessed by reusing the material over three consecutive cycles of photocatalytic degradation (20 min each) under visible light irradiation. As shown in Figure 13, glyphosate degradation efficiency decreased by only 5.03% after three cycles compared to the fresh material, indicating that the Ag_3_AsO_4_ photocatalyst exhibits high reusability in the degradation process and demonstrates potential as an excellent photocatalyst for oxidation reactions.

Despite the high stability and reusability demonstrated by Ag_3_AsO_4_ over three cycles, it is important to acknowledge a potential limitation regarding the chemical leaching of its constituent ions. The absence of quantitative data for silver and arsenic ions released into the aqueous medium during photocatalysis remains a critical factor. Considering the inherent toxicity of arsenic, further investigations are necessary to determine residual concentrations and ensure compliance with environmental safety standards. Therefore, the practical application of this photocatalyst for wastewater treatment must be preceded by a rigorous evaluation of ion leaching to mitigate potential secondary contamination risks.

#### 3.4.5. Photocatalytic Mechanism

To gain a deeper mechanistic understanding of the photocatalytic activity exhibited by Ag_3_AsO_4_, we analyzed its electronic band structure based on the experimental optical band gap energy (E_g_) of 1.55 eV, as determined by DRS analysis. We estimated the potentials of the valence band (E_VB_) and conduction band (E_CB_) by applying Mulliken electronegativity theory equations: $E_{VB}=x-E^{e}+0.5E_{g}$ and $E_{CB}=E_{VB}-E_{g}$, where x represents the absolute electronegativity of the semiconductor (ca. 6.06 eV for Ag_3_AsO_4_) and E^e^ is the energy of free electrons on the hydrogen scale (4.5 eV).

Application of these parameters yielded E_VB_ and E_CB_ values of 2.33 eV and 0.78 eV (vs. NHE), respectively. These findings correspond well with literature reports for silver arsenate, highlighting a valence band potential that is highly positive and thus capable of driving strong oxidation processes [30]. Upon irradiation with visible light (λ > 450 nm), electrons are excited to the conduction band, leaving holes in the valence band. Notably, the calculated E_CB_ (0.78 eV) is more positive than the standard redox potential for the oxygen/superoxide couple (O_2_/O_2_^−^•, −0.33 eV); consequently, photogenerated electrons are thermodynamically unable to reduce adsorbed O_2_ into superoxide radicals. Conversely, the E_VB_ potential of 2.33 eV is sufficient to enable photogenerated holes (h^+^) to either react with water/hydroxide ions to form hydroxyl radicals (•OH) or to directly oxidize organic contaminants.

In light of these energetic constraints, we propose that glyphosate removal over Ag_3_AsO_4_ proceeds primarily through direct oxidation by photogenerated holes (h^+^) and the involvement of hydroxyl radicals (•OH), leading to the formation of degradation products.

This mechanistic interpretation aligns with the insights provided by Tang et al. [30], reinforcing that the high oxidation capacity of valence band holes is the dominant driver of photocatalytic efficiency in this system.

### 3.5. Allium cepa Test

#### 3.5.1. Analysis of Cytotoxicity

The mitotic index allowed us to quantify the mitotic activity of meristematic cells in *Allium cepa* roots to estimate the cytotoxic potential of treatments. Glyphosate concentrations for treatments were established based on Brazilian drinking water standards, which set the maximum allowable level for this compound at 65 μg L^−1^ in freshwater for human consumption (Class 1 and 2) and 280 μg L^−1^ for Class 3 [47].

**Figure 14:**
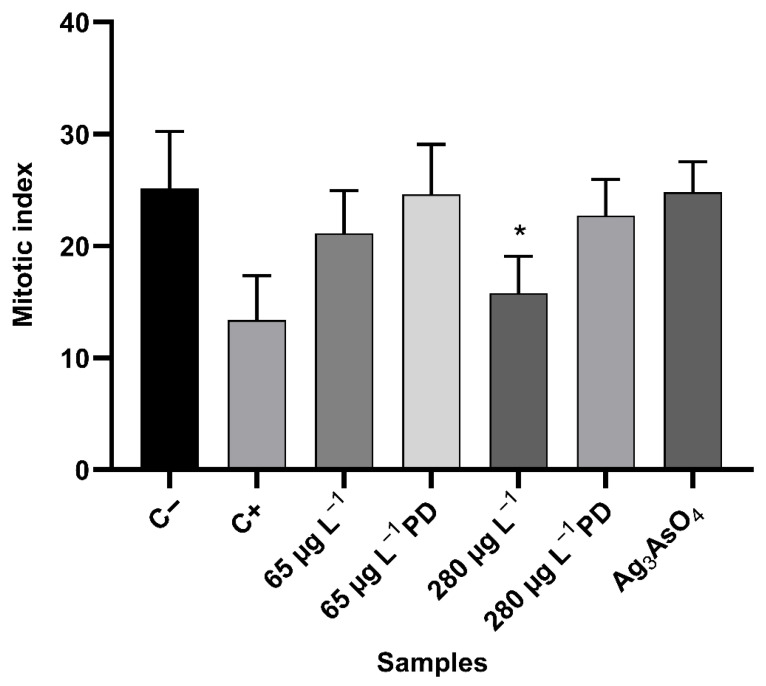
Mitotic index (mean ± standard deviation) of meristematic cells of *Allium cepa* exposed to controls and treatments with glyphosate and Ag_3_AsO_4_. PD: post-degradation. * *p* < 0.05 compared to the negative control.

Results for the mitotic index (mean ± standard deviation) and the total number of cells in interphase and cell division for each control and treatments are shown in Table 5. Figure 14 shows the arithmetic mean and standard deviation of the mitotic index for each sample.

Mann–Whitney test data show that the negative control differs significantly from the positive control (*p* = 0.0001). When comparing treatments before and after photodegradation, statistically significant differences were found between the 65 μg L^−1^ and 65 μg L^−1^ PD (after degradation) glyphosate treatments (*p* = 0.0450) and between the 280 μg L^−1^ and 280 μg L^−1^ PD treatments (*p* < 0.0001), with the mitotic index increasing after herbicide degradation, indicating the efficiency of the photodegradation process in reducing glyphosate concentration. There was no statistically significant difference between the negative control and Ag_3_AsO_4_, proving that the photocatalyst is not cytotoxic. It was observed that increasing glyphosate concentration in the treatments from 65 μg L^−1^ to 280 μg L^−1^ caused a decrease in the mitotic index, suggesting that exposure to this herbicide causes a dose-dependent decrease in mitotic activity. There was a statistically significant difference (*p* < 0.05) between the negative control and the treatment with glyphosate at 280 μg L^−1^ (*p* = 0.0004), indicating the cytotoxic potential of this herbicide.

The cytotoxicity of glyphosate can be attributed to its ability to cause oxidative stress in plant cells, which accelerates lipid peroxidation and impairs cell membranes integrity. Glyphosate can increase reactive oxygen species levels, generating free radicals that damage cell proteins and DNA in excess, leading to cytotoxicity in plants. In addition, glyphosate can decrease the synthesis of and bind to plant tubulin protein, inhibiting microtubule polymerization, which is responsible for chromosome migration during cell division [48,49].

#### 3.5.2. Analysis of Genotoxicity

**Figure 15:**
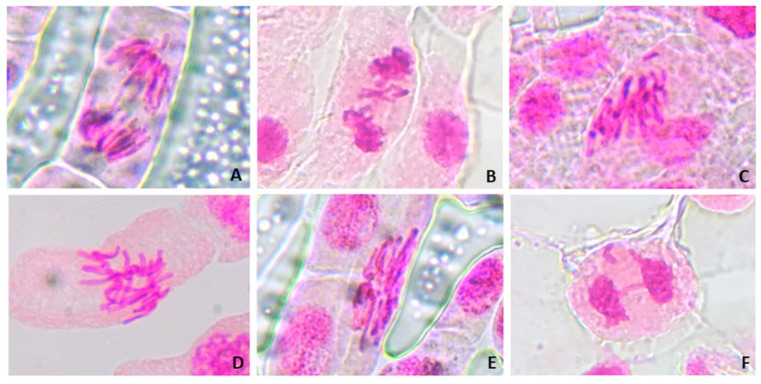
Light micrographs of chromosomal aberrations in meristematic cells of *Allium cepa* exposed to controls and treatments with glyphosate and Ag_3_AsO_4_. (**A**): anaphase with chromosomal bridge; (**B**): telophase with chromosomal loss; (**C**): metaphase with chromosomal breakage; (**D**): polyploid metaphase; (**E**): metaphase with chromosomal adherence (stickiness); (**F**): telophase with laggard chromosome and breakage. Final magnification of 100×.

**Figure 16:**
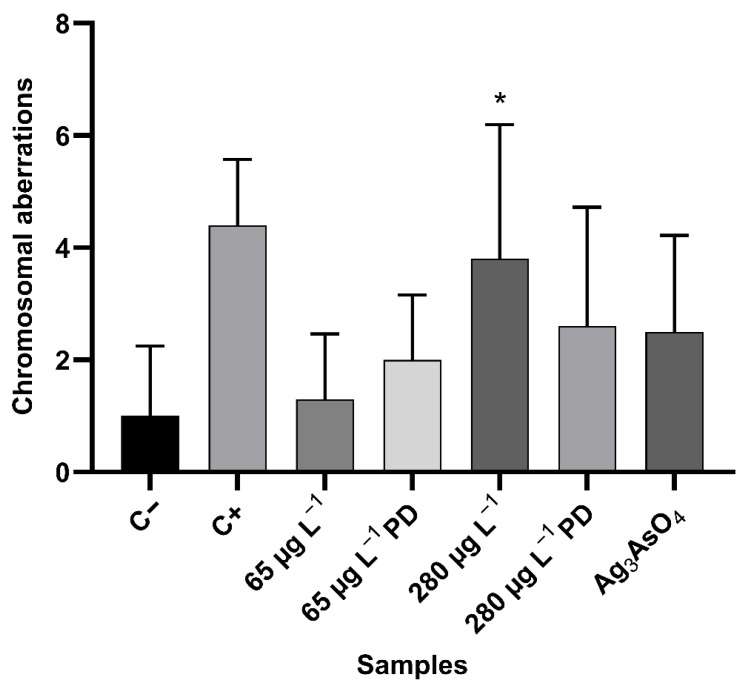
Number of chromosomal aberrations (mean ± standard deviation) in meristematic cells of *Allium cepa* exposed to controls and treatments with glyphosate and Ag_3_AsO_4_. PD: post-degradation. * *p* < 0.05 compared to the negative control.

The genotoxicity study was performed by analysing chromosomal aberrations in meristematic cells of *Allium cepa* roots exposed to glyphosate and Ag_3_AsO_4_. Alterations considered for this study were: bridge, chromosomal loss, chromosomal breakage, polyploid metaphase, metaphase with chromosomal adherence (stickiness), and laggard chromosome (Figure 15). Results regarding the type and frequency of abnormalities in these cells for each control and treatment are presented in Table 6. Figure 16 shows the arithmetic mean and standard deviation of chromosomal changes for each sample.

Using the Mann–Whitney test results, a statistically significant difference was found between the negative and positive control (*p* < 0.0001). There was no statistically significant difference between the negative control and Ag_3_AsO_4_, indicating that the material has no genotoxic potential. It was observed that the frequency of total chromosomal aberrations increased with increasing glyphosate concentration from 65 μg L^−1^ to 280 μg L^−1^, suggesting that exposure to this herbicide is associated with a dose-dependent increase in chromosomal alterations. There was a statistically significant difference (*p* < 0.05) in chromosomal abnormalities only between the negative control and the 280 μg L^−1^ treatment (*p* = 0.0071). These results indicate that glyphosate acts directly on genetic material and therefore has a genotoxic potential.

Chromosomal abnormalities are characterized by changes in the structure or total number of chromosomes at different stages of cell division and can occur spontaneously or as a result of exposure to contaminants such as glyphosate. The development of glyphosate-induced chromosomal aberrations may be related to the direct interaction of glyphosate with DNA. This interaction can lead to DNA dissociation, violation of integrity, chain breaks, micronuclei formation, and chromosomal aberrations, explaining the genotoxicity of this herbicide [49,50,51].

#### 3.5.3. Analysis of Mutagenicity

**Figure 17:**
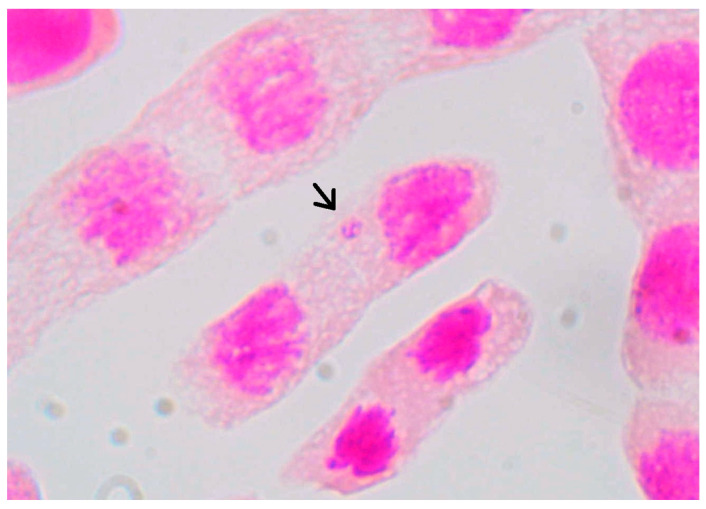
Light micrograph with an arrow indicating the presence of a micronucleus in a meristematic cell of *Allium cepa* exposed to glyphosate treatment. Final magnification of 100×.

**Figure 18:**
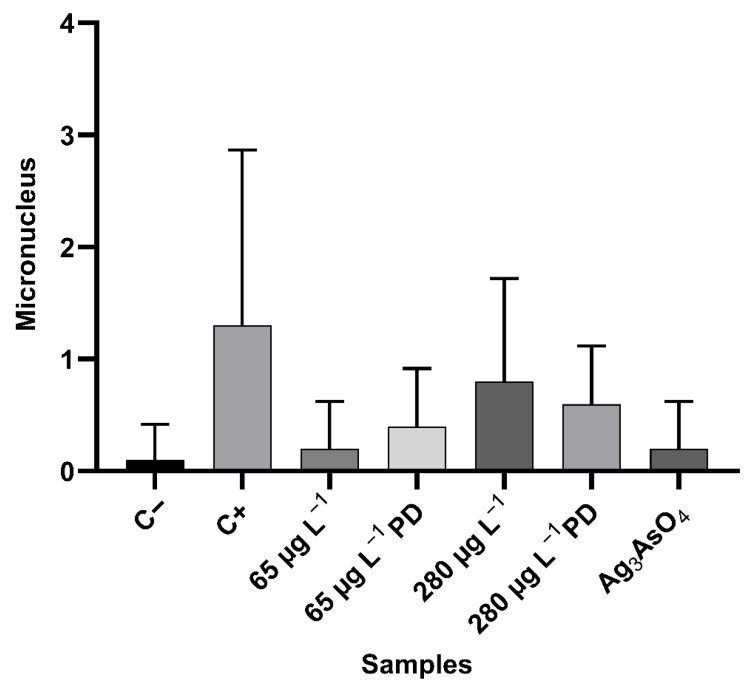
Number of micronuclei (mean ± standard deviation) in meristematic cells of *Allium cepa* exposed to controls and treatments with glyphosate and Ag_3_AsO_4_. PD: post-degradation.

Micronuclei are small structures consisting of chromatin enveloped by a nuclear membrane. They are formed by chromosome lagging during cell division or by DNA degradation [52]. The mutagenic potential of glyphosate and Ag_3_AsO_4_ was assessed by analyzing the frequency of micronuclei in meristematic cells of *Allium cepa*. This analysis was performed for all phases of the cell cycle (interphase, prophase, metaphase, anaphase, and telophase). Figure 17 shows the presence of a micronucleus in a prophase cell. Data on the occurrence of micronuclei in the samples examined are listed in Table 7. Figure 18 shows the arithmetic mean and standard deviation of mutagenicity anomalies for each sample.

Data obtained with the Mann–Whitney test showed a statistically significant difference between the negative and positive control (*p* = 0.0263). It was observed that as glyphosate concentration increased in the treatments from 65 μg L^−1^ to 280 μg L^−1^, the occurrence of micronuclei increased, suggesting that exposure to this herbicide causes a dose-dependent increase in micronuclei frequency. There was no statistically significant difference (*p* < 0.05) between the negative control and glyphosate or Ag_3_AsO_4_ treatments, suggesting that they have no mutagenic potential at the tested concentrations.

Although chromosomal aberrations (CAs) were observed, indicating genotoxic activity, no significant induction of micronuclei (MN) was detected. This discrepancy can be attributed to the kinetics of the cell cycle and the exposure duration. Chromosomal aberrations reflect immediate damage occurring during mitosis, whereas micronuclei are formed from acentric fragments or lagging chromosomes that are excluded from the main nucleus in the subsequent interphase. Therefore, the short exposure time to glyphosate may have been insufficient for the formation of stable micronuclei, or the severity of the damage may have led to cell death (cytotoxicity), preventing the fixation of the damage as a permanent mutation [34,35].

### 3.6. MTT Assay

**Figure 19:**
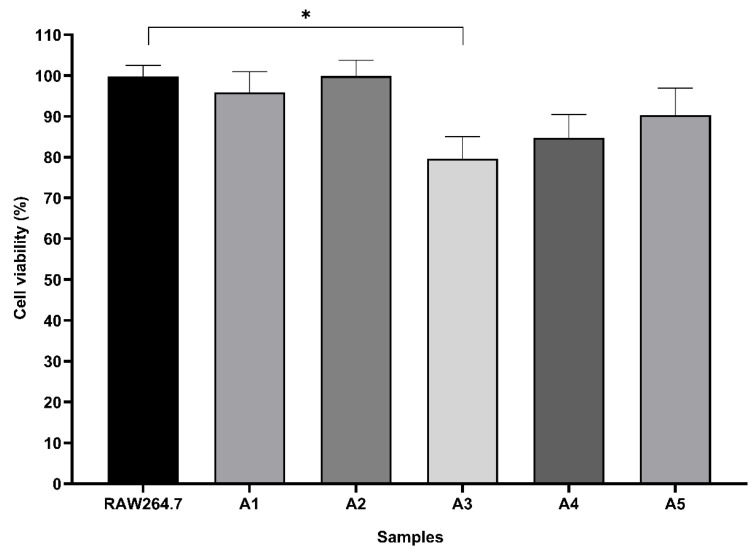
Cell viability analyzed using the MTT method. RAW264.7: untreated cells (negative control); A1: cells treated with standard glyphosate solution at 65 μg L^−1^; A2: cells treated with standard glyphosate solution at 65 μg L^−1^ after degradation; A3: cells treated with standard glyphosate solution at 280 μg L^−1^; A4: cells treated with standard glyphosate solutions at 280 μg L^−1^ after degradation; A5: cells treated with Ag_3_AsO_4_ at 0.1% (*w*/*v*). * Influenced by cytotoxicity. Representative data from 3 independent experiments.

Figure 19 shows the cell viability of RAW264.7 macrophages. Data show that cell viability was greater than 80% for all treatment, except for the treatment with glyphosate solution at 280 μg L^−1^ (A3), which showed a statistically significant difference compared to the negative control (*p* < 0.05) and is considered cytotoxic to macrophage cells. In addition, higher cellular viability is observed in macrophages treated with glyphosate solutions after degradation with Ag_3_AsO_4_ (A2 and A4) compared to standard solutions of the same concentration (A1 and A3), which is attributed to glyphosate removal by the photodegradation reaction. Cells treated with Ag_3_AsO_4_ (A5) showed high cell viability, indicating low cytotoxicity of the material under tested conditions. Thus, results indicate that glyphosate has a cytotoxic effect and affects the RAW264.7 cell cycle of in a dose-dependent manner.

According to resolutions established by CONAMA [47], the maximum allowable level for glyphosate in freshwater for human consumption (Class 3, conventional or advanced treatment) is 280 μg L^−1^, and the maximum residue limit for glyphosate plus AMPA in groundwater for human consumption is 500 μg L^−1^. However, results from the MTT assay and the *Allium cepa* test demonstrated cytotoxicity at the concentration of 280 µg L^−1^. Consequently, these findings indicate that glyphosate exhibits cytotoxic potential even at the regulatory limit of 280 µg L^−1^. These results reinforce the importance of continuous toxicological monitoring and further investigation to ensure the safety of water intended for human consumption.

### 3.7. Quantification of Nitric Oxide

**Figure 20:**
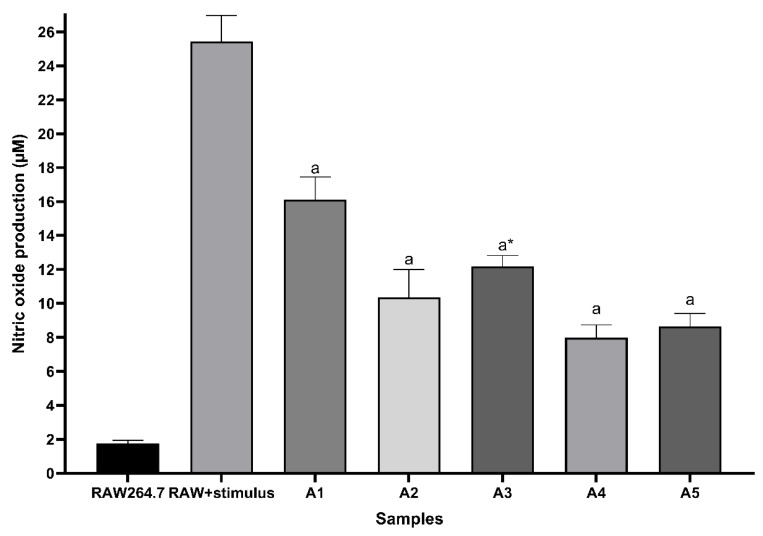
Nitric oxide production in RAW264.7 cells stimulated by LPS. RAW264.7: negative control (untreated and unstimulated macrophages); RAW + stimulus: positive control (LPS-stimulated and untreated macrophages); A1: cells treated with standard glyphosate solution at 65 μg L^−1^; A2: cells treated with standard glyphosate solution at 65,0 μg L^−1^ after degradation; A3: cells treated with standard glyphosate solution at 280 μg L^−1^; A4: cells treated with standard glyphosate solutions at 280 μg L^−1^ after degradation; A5: cells treated with Ag_3_AsO_4_ at 0.1% (*w*/*v*). a: *p* < 0.05 compared with RAW + stimulus cells. * Influenced by cytotoxicity. Representative data from 3 independent experiments.

Nitric oxide concentration was determined indirectly by quantifying nitrite in the crop supernatant using the Griess method. Figure 20 shows nitric oxide production in RAW264.7 cells. Results show that all treatments inhibited nitric oxide production in lipopolysaccharide-stimulated (LPS) RAW264.7 macrophages. There were statistically significant differences (*p* < 0.05) compared to the result obtained in the positive control (RAW + stimulus) macrophages. Treatments with Ag_3_AsO_4_ and standard glyphosate solutions after degradation achieved better results in reducing nitric oxide production.

However, for the treatment with glyphosate at 280 µg L^−1^, the decrease in nitric oxide levels parallels the reduction in cell viability observed in the MTT assay. Thus, the decrease in nitric oxide production at this concentration is likely a consequence of cytotoxicity rather than a distinct inhibitory mechanism.

## 4. Conclusions

This work successfully validated a rapid and sensitive analytical method for glyphosate quantification and demonstrated the efficacy of Ag_3_AsO_4_ as a visible-light photocatalyst. The proposed derivatization method achieved detection limits well below maximum thresholds established by national and international regulatory agencies, ensuring its applicability for monitoring water intended for human consumption.

Glyphosate photodegradation in water with Ag_3_AsO_4_ showed a removal efficiency of 99.46% after 60 min of visible light irradiation. This result indicates the photocatalytic potential of Ag_3_AsO_4_ under visible light, making it a promising material for wastewater treatment to improve water quality and reduce potential human health risks associated with this pollutant. However, it is important to note that this study focused on the removal of the parent compound, and its full-scale application requires further evaluation of ion stability, identification of transformation products (e.g., AMPA), and mineralization rates using Liquid Chromatography–Mass Spectrometry (LC-MS) and Total Organic Carbon (TOC) analyses, as well as leaching assessments to ensure environmental safety.

The *Allium cepa* test showed positive results for glyphosate cytotoxicity and genotoxicity, which were confirmed by the MTT assay, where glyphosate showed cytotoxic potential for macrophage cells at a regulatory limit of 280 μg L^−1^. These findings suggest that the presence of glyphosate at this concentration may pose potential biological risks, highlighting the need for stringent surveillance and comprehensive risk assessment regarding water contamination.

Finally, it is worth noting that experiments in this study were conducted using laboratory-prepared standard solutions. This approach was chosen to isolate the photocatalytic mechanism and ensure analytical validation robustness, precluding the interference of complex matrix effects. While these results lay a solid foundation as a proof of concept, future studies are required to evaluate photocatalyst performance in real-world environmental matrices, such as agricultural runoff and river water, to fully assess the influence of co-existing ions and natural organic matter, while strictly evaluating metal leaching to ensure environmental safety.

## Figures and Tables

**Table 1:** Parameters of the regression equation of the proposed HPLC-FLD method for the determination of glyphosate.

Parameters	Values
Determination coefficient (R^2^)	0.99954
Correlation coefficient (R)	0.99976
Slope (a)	2.06531 × 10^8^
Y-intercept (b)	4.49216 × 10^8^
Concentration range (µg L^−1^)	1–100

**Table 2:** Glyphosate precision test results (*n* = 3).

Concentration (µg L^−1^)	RSD (%)	
	Repeatability	Intermediate Precision
1	1.0546	2.4127
5	4.7209	5.6227
12.5	6.2865	9.0462

RSD: relative standard deviation.

**Table 3:** Results of the glyphosate recovery study.

Concentration (µg L^−1^)	Measured Concentration (Mean ± SD) (µg L^−1^)	Recovery (%)
1	0.99 ± 0.01	99.41
5	4.69 ± 0.22	93.84
12.5	11.96 ± 0.75	95.73

SD: standard deviation.

**Table 4:** Comparison of glyphosate photodegradation efficiency using different catalysts reported in the literature.

Photocatalyst	Irradiation Time (min)	Photodegradation Efficiency (%)	References
TiO_2_	210	92.00	[39]
MoSe_2_/BiVO_4_	180	86.10	[40]
BiOBr/Fe_3_O_4_	60	97.00	[41]
CuS/Bi_2_WO_6_	180	85.90	[42]
In_2_S_3_/BiVO_4_	180	74.66	[43]
GQDs/TNAs	60	94.70	[44]
Co_3_O_4_/BiOBr	120	88.50	[45]
C-TiO_2_/clinoptilolite	30	84.00	[46]
Ag_3_AsO_4_	60	99.46	This study

**Table 5:** Number of meristematic cells of *Allium cepa* in interphase, division, and mitotic indices (mean ± standard deviation) of controls and treatments with glyphosate and Ag_3_AsO_4_.

Samples	Total Cells Analyzed	Cells in Interphase	Cells in Mitosis	Mitotic Index (Mean ± SD)
C−	5000	3743	1257	25.14 ± 4.11
C+	5000	4331	669	13.38 ± 3.38
65 µg L^−1^	5000	3944	1056	21.12 ± 3.30
65 µg L^−1^ PD	5000	3770	1230	24.60 ± 3.36
280 µg L^−1^	5000	4211	789	15.78 ± 2.79
280 µg L^−1^ PD	5000	3865	1135	22.70 ± 2.82
Ag_3_AsO_4_	5000	3761	1239	24.78 ± 2.26

SD: standard deviation; PD: post-degradation.

**Table 6:** Frequency of chromosomal aberrations in meristematic cells of *Allium cepa* exposed to controls and treatments with glyphosate and Ag_3_AsO_4_.

Samples	Bridge	Loss	Breakage	Polyploid Metaphase	Stickiness	Laggard Chromosome	Total
C−	2	0	0	3	5	0	10
C+	8	2	2	12	18	2	44
65 µg L^−1^	5	0	0	2	5	1	13
65 µg L^−1^ PD	6	0	1	6	13	0	26
280 µg L^−1^	7	1	2	6	19	3	38
280 µg L^−1^ PD	7	1	2	5	15	1	31
Ag_3_AsO_4_	5	0	0	7	13	0	25

**Table 7:** Frequency of micronuclei in meristematic cells of *Allium cepa* exposed to controls and treatments with glyphosate and Ag_3_AsO_4_.

Samples	Total Cells Analyzed	Micronucleus
C−	5000	1
C+	5000	13
65 µg L^−1^	5000	2
65 µg L^−1^ PD	5000	4
280 µg L^−1^	5000	8
280 µg L^−1^ PD	5000	6
Ag_3_AsO_4_	5000	2

PD: post-degradation.

## Data Availability

Data are contained within the article.
